# Late Onset Atraumatic Ceramic Head Fracture of a Hybrid Ceramic Bearings Total Hip Arthroplasty

**DOI:** 10.7759/cureus.13726

**Published:** 2021-03-06

**Authors:** Ioannis Papaioannou, Thomas Repantis, Georgia Pantazidou, Andreas Baikousis, Panagiotis Korovessis

**Affiliations:** 1 Orthopedics, General Hospital of Patras, Patras, GRC; 2 Otolaryngology - Head and Neck Surgery, General Hospital of Patras, Patras, GRC

**Keywords:** hip arthroplasty, hybrid, ceramic bearings, head fracture, revision

## Abstract

Ceramic head fracture is a major complication of ceramic-on-ceramic (CoC) total hip arthroplasty (THA) and though new generation ceramics have lowered the rates, although it is still a great concern. We report a case of late onset (more than 10 years after surgery) ceramic head fracture of a hybrid ceramic bearings to emphasize on its unusual clinical manifestation. Furthermore, we highlight the late onset presentation and also the rarity of this complication with this particular hybrid ceramic bearings. A relevant review of the literature revealed that hybrid ceramic bearings need to be more thoroughly studied to understand modes of their failure and to reach a consensus on how to reduce and prevent these disastrous complications.

## Introduction

Ceramic-on-ceramic (CoC) total hip arthroplasty (THA) has gained popularity due to its mechanical characteristics, despite the concerns reported about fracture of the ceramic components and especially the femoral head [1,2]. During the development of Delta ceramics, there was a period in which some surgeons used Delta ceramic liner (fourth generation ceramic) and alumina femoral head (third generation ceramic). The evidence for the long-term results of this hybrid ceramic coupling is limited. We report a unique case of a non-traumatic late onset fracture of an alumina femoral head coupled with a Delta ceramic liner. To the best of our knowledge, this is the first alumina femoral head fracture articulating with Delta liner and the fourth report of a late onset (more than 10 years postoperatively) atraumatic ceramic head fracture [3-5].

## Case presentation

**Figure 1:**
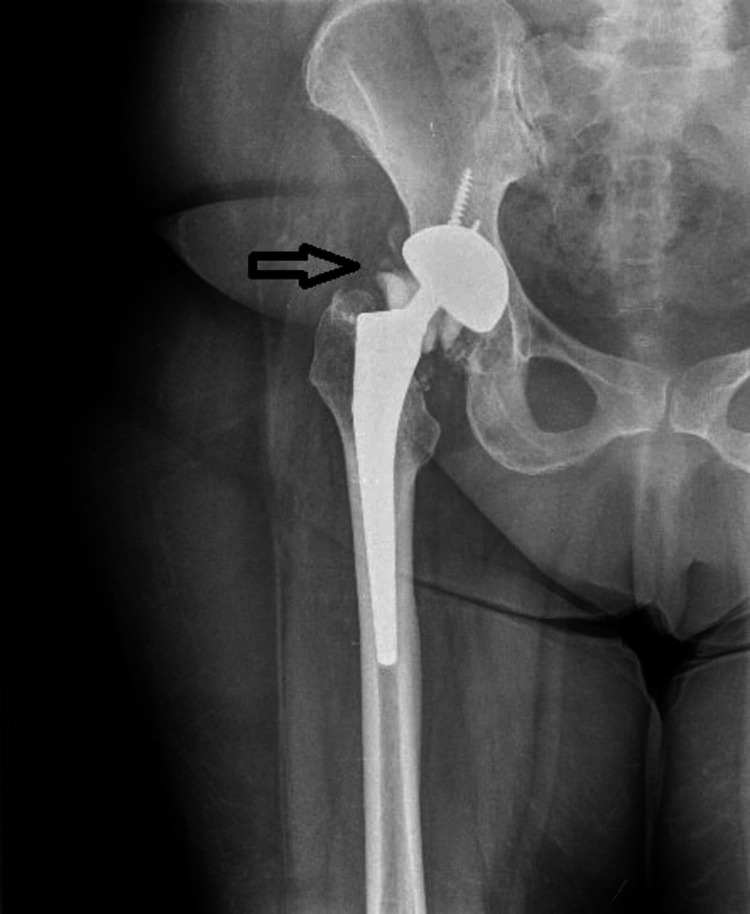
Anteroposterior hip radiograph demonstrates the fractured femoral head (black arrow). No osteolysis is presented.

A 71-year-old female patient presented to our outpatient clinic reporting difficulty in walking due to mild pain in her right hip and an audible annoying cracking sound coming from her ipsilateral knee for the last 10 days. She reported no fall or major trauma. Her body mass index (BMI) was 30.2 and she was active with minor comorbidities (only idiopathic hypertension). Clinical examination revealed leg length discrepancy, mild pain in her right groin during flexion and rotation of the right hip, whereas movements of the right knee were normal and painless. The patient underwent ceramic-on-ceramic right THA 11 years ago, due to primary osteoarthritis. Radiographic evaluation of the right hip was indicative for ceramic head fracture, while no osteolysis was observed (Figure 1).

**Figure 2:**
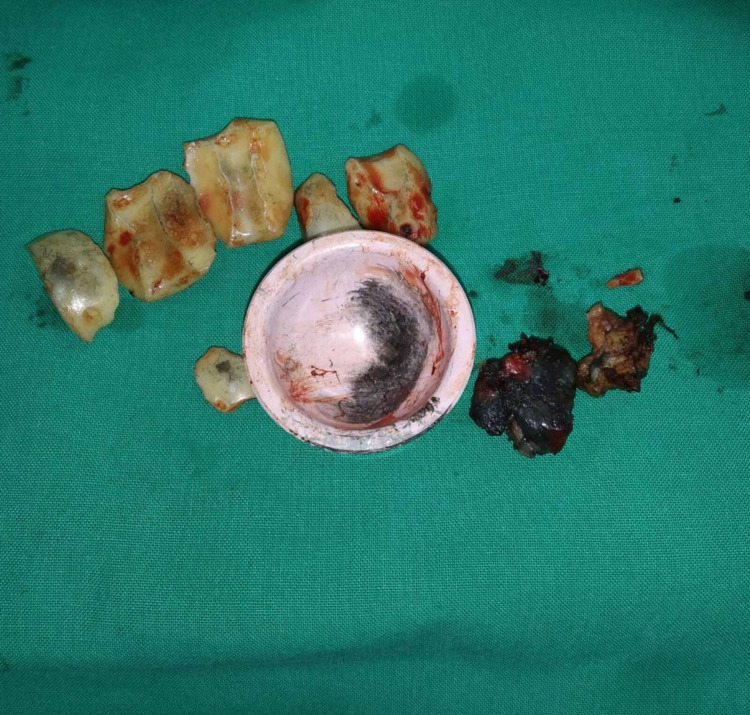
Intraoperative photo of the fractured alumina head, the worn delta ceramic liner and a part of the excised soft tissue after the debridement.

A press fit THA (Premedical Jump System) with hybrid ceramic bearings had been implanted to the patient. The head was 28-mm third-generation ceramic (alumina, Biolox Forte, CeramTec, Plochingen, Germany), while the liner was fourth generation ceramic (Biolox Delta, CeramTec, Plochingen, Germany). The patient underwent revision surgery two days after admission. All the fragments of the ceramic head were removed, all the affected tissues were excised, and the ceramic insert of the acetabular shell was also exchanged because it was found worn-out (Figure 2).

**Figure 3:**
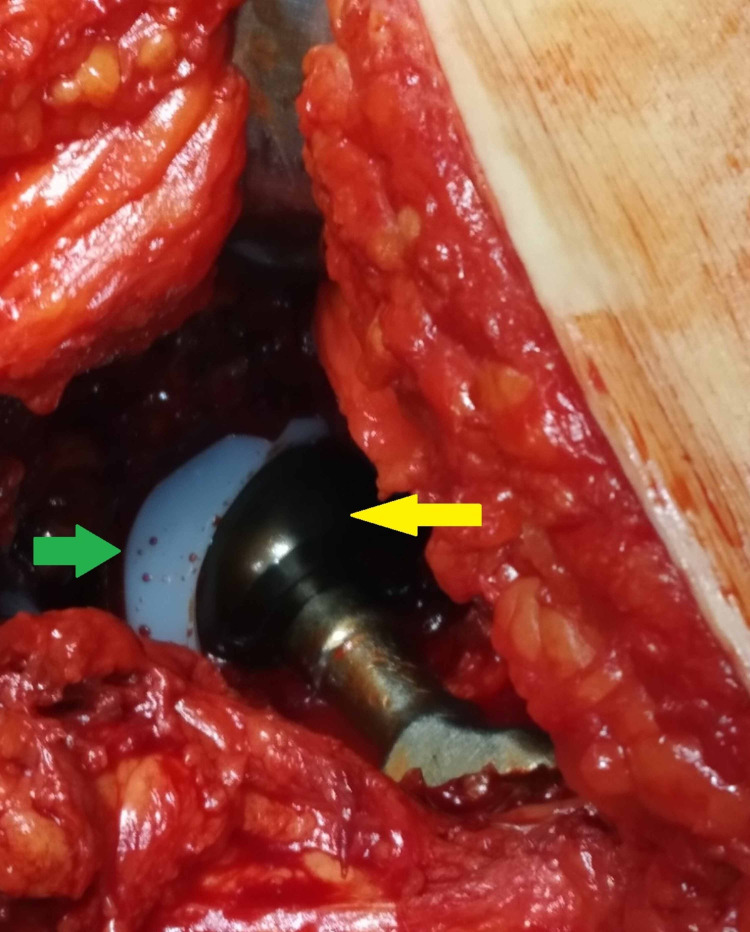
Intraoperative photo after the revision of the hybrid ceramic bearings. The green arrow indicates the highly cross-linked polyethylene (HXLPE) and the yellow arrow indicates the oxidized zirconium (Oxinium, Smith & Nephew) head.

The stem and the acetabular shell were found stable and the trunnion was in satisfactory condition to retain the femoral component. We implanted an oxidized zirconium head (Oxinium, Smith & Nephew, London, UK) and a highly cross-linked polyethylene (HXLPE) (Figure 3).

**Figure 4:**
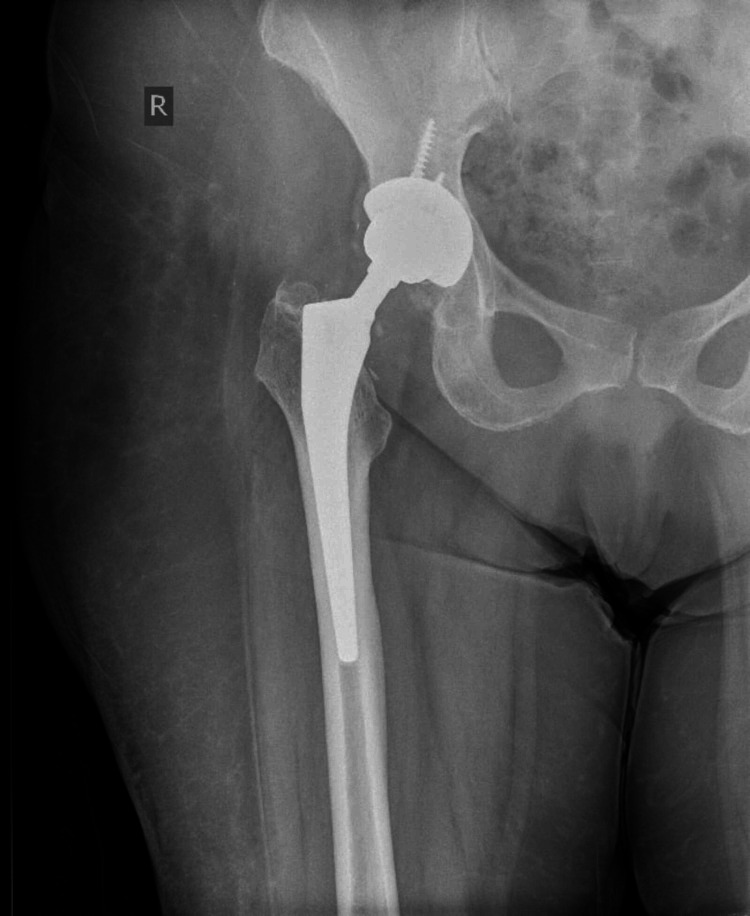
Postoperative hip radiograph after the revision of the ceramic bearings.

The patient had an uneventful recovery, the radiographic evaluation was satisfactory (Figure 4) and thus the patient was mobilized on the first postoperative day with full weight bearing.

At the last follow-up, 12 months postoperatively she had returned to her previous activity with excellent function (Harris Hip Score: 91/100) and without any complaints.

## Discussion

The generation of the ceramics plays a crucial role to the reported incidence of ceramic component's fracture rate. First and second generation ceramics were associated with increased fracture rate (13.4%), while this rate has been reduced dramatically to 0.1% for third generation ceramics and to 0.001% for Biolox Delta ceramics [2,3]. The main risk factors for ceramic head fractures could be the following: obesity, high activity level, trauma, small ball diameter, poor manufacturing techniques and poor surgical technique [1]. The development of the third and fourth generation ceramics has led to significant reduction of the fracture rate (even 0.001%) [2]. Delta ceramic includes 24% of Zirconia and has been developed to reduce the rate of ceramic fracture [6]. This was the main difference between Delta ceramics and alumina ceramics. Recent data highlighted that the fourth generation of ceramics have reduced the odds of head fracture, but not that of liner, because Biolox Forte and Biolox Delta liners have similar liner fracture incidence, 0.112% and 0.126% respectively [1]. This occurs due to the specific mechanism of the ceramic liner fracture, which is associated with impingement between the neck of the femoral stem and the anterior rim of the acetabular component [7].

According to a large metanalysis, the majority (83%) of the fractures of the ceramic components occur within 36 months after implantation. Almost all the reported ceramic femoral head fractures happened within the first six years postoperatively [2]. Late onset (more than 10 years postoperatively) ceramic femoral head fracture with no trauma history is a rare entity with only three cases published. Two of them concern ceramic head fracture with ceramic-on-polyethylene (CoP) articulation [4,5] and only one with CoC bearing [3], as in our case. To the best of our knowledge, this is the first report of a fracture of an alumina femoral head coupling with a delta ceramic liner, the so-called hybrid bearing. So far, there were only two studies dealing with this combination of bearing surfaces, with no report of an alumina head fracture [6,7]. In an effort to explain this catastrophic event in our case, we have recognized the following potential risk factors. Our patient was obese and active, and the femoral head implanted in the primary THA was 28 mm. The use of 28-mm head and shorter neck intraoperatively were established risk factors for component breakage [1,2]. Hybrid ceramic bearing articulation may promote long-term wear via the low temperature degradation of zirconia articulating with alumina and this should be taken into consideration among hip arthroplasty surgeons. The suggested mechanism of the failure of this hybrid ceramic coupling is the transformation of tetragonal phase zirconia (component of Delta ceramics) to low energy monoclinic phase, the so-called low temperature degradation. This transformation can result to lower hardness and lower resistance to crack formation compared to the tetragonal phase, increased fracture incidence and also increased surface roughness, ceramic wear and osteolysis [6-8]. Revision strategy concerning the new bearings after the ceramic femoral head fracture is still controversial, although there is consensus that if the trunnion is not salvageable, the revision of the stem is indicated [1]. Furthermore, the results of metal head on polyethylene liner implantation after ceramic head fracture were disappointed and unacceptable with severe metallosis and even pseudotumor formation [9] due to increased third body wear from the remaining microscopic ceramic fragments [1]. Ceramic-on-ceramic or ceramic-on-polyethylene seems to eliminate the risk for third body wear due to the elevated scratch resistance of the ceramics, although there is no consensus about the best articular coupling [10].

## Conclusions

Ceramic head fracture should be included in the differential diagnosis of acute or subacute groin pain after CoC THA, even with late onset of presentation. As this case concerned a unique late onset alumina head fracture coupling with Delta ceramic liner, this hybrid articulation should alert surgeons for meticulous follow-up.
